# Proactive home-based malaria management in rural communities of Bassar Health District in northern Togo from 2014 to 2017: PECADOM + , a pilot experiment

**DOI:** 10.1186/s12936-024-04988-x

**Published:** 2024-07-07

**Authors:** Tchaa A. Bakai, Maë Gense, Philippe Vanhems, Jean Iwaz, Anne Thomas, Tinah Atcha-Oubou, Tchassama Tchadjobo, Nicolas Voirin, Nagham Khanafer

**Affiliations:** 1 Epidemiology and Modelling in Infectious Diseases (EPIMOD), 01240 Lent, France; 2 Programme National de Lutte Contre le Paludisme (PNLP), 01 BP 518, Lomé, Togo; 3 Équipe Santé Publique, Épidémiologie et Écologie Évolutive des Maladies Infectieuses (PHE3ID), Centre International de Recherche en Infectiologie (CIRI), Institut National de la Santé et de la Recherche Médicale (INSERM U1111), Centre National de la Recherche Scientifique (CNRS UMR 5308), École Normale Supérieure de Lyon, Université Claude-Bernard, Lyon 1, Lyon, France; 4 Service d’Hygiène, Épidémiologie et Prévention, Hôpital Édouard Herriot, Hospices Civils de Lyon, 69003 Lyon, France; 5 SCI & MED ED, Lyon, France

**Keywords:** Proactive screening, Community care, Home care, Malaria, Togo, Rapid diagnostic test

## Abstract

**Background:**

Togo's National Malaria Control Programme has initiated an active home-based malaria management model for all age groups in rural areas of Bassar Health District. This report describes the model, reports its main results, and determines the factors associated with positive rapid diagnostic test results.

**Methods:**

From 2014 to 2017, in three peripheral care units of Bassar Health District (Binaparba, Nangbani, and Baghan), community health workers visited residents' homes weekly to identify patients with malaria symptoms, perform rapid diagnostic tests in symptomatic patients, and give medication to positive cases. Univariate and multivariate logistic regression models were used to determine the factors associated with positive tests.

**Results:**

The study covered 11,337 people (817 in 2014, 1804 in 2015, 2638 in 2016, and 6078 in 2017). The overall mean age was 18 years (95% CI 5–29; min–max: 0–112 years). The median age was 10 years (SD: 16.9). The proportions of people tested positive were 75.3% in Binaparba, 77.4% in Nangbani, and 56.6% in Baghan. The 5–10 age group was the most affected category (24.2% positive tests). Positive tests were more frequent during the rainy than during the dry season (62 vs. 38%) and the probability of positive test was 1.76 times higher during the rainy than during the dry season (adjusted OR = 1.74; 95% CI 1.60–1.90). A fever (37.5 °C or higher) increased significantly the probability of positive test (adjusted OR = 2.19; 95% CI 1.89–2.54). The risk of positive test was 1.89 times higher in passive than in active malaria detection (adjusted OR = 1.89; 95% CI 1.73–2.0).

**Conclusions:**

This novel experimental community and home-based malaria management in Togo suggested that active detection of malaria cases is feasible within 24 h, which allows rapid treatments before progression to often-fatal complications. This PECADOM + program will help Togo's National Malaria Control Programme reduce malaria morbidity and mortality in remote and hard-to-reach communities.

## Background

Malaria is one of the greatest threats to human life in developing countries [1] and, in most African countries, malaria is the leading cause of death, especially in children. According to the United Nations Children's Fund (UNICEF), a child dies from malaria every 30 s [2]. In the latest World Malaria Report issued by the World Health Organization (WHO), the estimated number of malaria deaths was 619,000, of which 96% occurred in Africa [3]. At the peak of the COVID-19 pandemic (2020–2021), COVID-19-related disruption to malaria control resulted in nearly 13 million additional malaria cases and 63,000 additional malaria deaths [3]. In terms of expenditure, the annual cost of malaria in Africa was 12 billion US dollars [1].

Early and correct management of malaria cases within 24 h of symptom onset has always been a concern for "Roll Back Malaria" partners and the WHO [4–9]. The WHO reiterated this strategic principle in its latest Global Malaria Action Plan, urging countries to take initiatives to guarantee malaria control services to populations in areas without health facilities by promoting home-based management of malaria cases: programme “Prise en Charge à Domicile” (PECADOM) or home care [4–6]; i.e., “an array of health and social support services provided to clients in their own residence”. Since the Declaration of Alma-Ata (1978), several countries have involved community health workers (CHWs) in supporting access to primary health care in remote or deprived communities [10].

CHWs are non-medical volunteers or paid members of a community, who are able to offer early and local access to certain healthcare services. Regarding malaria, CHWs help reduce the disease burden‒transmission, morbidity, and mortality by means of: (i) case management; i.e., diagnosis with rapid diagnostic tests (RDTs), treatment of fever and uncomplicated malaria cases, and referral of complicated cases to health facilities; (ii) prevention; i.e., education about malaria and its complications, intermittent preventive treatment for pregnant women or children, and supply of insecticide-treated bed nets; and, (iii) collection of key epidemiological data needed for surveillance and control. The actions of CHWs proved successful, provided sufficient funding and remuneration, adequate training, clear role definitions and guidelines, regular supervision, constant support, motivating incentives, and better recognition by the healthcare system (and thus the community itself) [11].

In Africa, Senegal has led the way with various community-based initiatives [1]. In 2009, Senegal extended its community-based programmes to villages more than five km from a health facility [12]. Based initially on a passive case detection model, this initiative significantly reduced malaria-related deaths. In this passive model, community members sought treatment from CHWs only when they suspected malaria [12]. In 2012, to remedy the main weakness of that passive model (i.e., waiting for the patients to seek care), an active case detection model (named PECADOM +) was initiated in Kédougou Region where CHWs circulated and searched for malaria cases in homes [12]. The success of this new model led to its adoption by the US Presidents of Malaria Initiative (in partnership with the Senegalese Ministry of Health) and its extension to the whole of Senegal [12].

In Togo, activity decentralization and community involvement in health actions are paradigms already adopted by the Ministry of Health and Public Hygiene to optimize the results of anti-malarial interventions. After discussions with its financial and technical partners, the Togolese authorities approved PECADOM + for implementation as part of its National Malaria Strategic Plan (NMSP). However, a pilot study–limited to a part of the country–had to be carried out to evaluate the feasibility and the outcomes of the project as well as the involvement of the local actors, authorities, and beneficiaries. During this study, a full adhesion to the project was observed and a great satisfaction was expressed by the actors, the partners, and the beneficiaries.

Within this framework, inspired by the Senegalese experience, and in collaboration US Peace Corps Togo, the National Malaria Control Programme (NMCP) implemented, between 2014 and 2017, a community-based model of active malaria screening and treatment in villages located more than five km from a Peripheral Care Unit (PCU) in the Health District of Bassar (Kara Region of Northern Togo). The choice of the region was based on data showing that Bassar had the highest rate of malaria transmission [13]. Currently, the Togolese PECADOM + project is active in 17 villages, employs 21 CHWs, and reaches nearly 6,000 people.

This study aimed to describe this malaria active screening model, show its main results, and determine the factors associated with positive rapid diagnostic tests in Bassar communities.

## Methods

### PECADOM + in Togo

In Togo, this pilot model was set up between 2014 and 2017 after an introductory feasibility study in Bassar communities. For this feasibility study and set up, the following methodological steps were used:

- *Documentary research work*: this work involved the analysis of several documents: the project protocol, workshop reports, training and coordination reports, information documents on the Bassar health district (in particular, the malaria situation), and the characteristics of the participating villages (including the number of inhabitants, distance from the reference health facility).
- *Interviews:* these were carried out with stakeholders such as NMCP technical managers, project partners, department managers, local administrative authorities, community leaders, and benefiting communities.
- *Site visits*: several candidate villages in Bassar Health District were visited to meet local stakeholders and agree with them on the project outline.
- *Selection of the participating villages*: after an orientation workshop held with all the stakeholders, these villages were selected on of the following criteria:
  o Being located in the Bassar Health District area;
  o Being deprived of an appropriate health infrastructure;
  o Being temporarily or permanently located within five km from the nearest health facility (difficult-to-reach areas, islands, disaster zones).
- *Selection of the CHWs*: these workers were selected according to the following criteria:
  o Being residents in their respective villages;
  o Being volunteers;
  o Being nominated by their respective communities;
  o Being available and committed to each community's interests;
  o Being able to read and write in French and speak local languages.
- *Training of the CHWs*: this included a theoretical and a practical step. The training focused on malaria transmission, recognition of the signs of uncomplicated and severe malaria, use of RDTs with strict adherence to hygiene rules, treatment with an artemisinin-based combination therapy (ACT), reasons for case referral, and maintenance of management tools (registers, stock sheets, monthly reports).
- *Supervision, monitoring, and evaluation of the strategy*: these actions were carried out at various levels by NMCP managers, regional managers, district managers, and managers of the PCU.

### PECADOM + experimental study environment

Togo is a West African country with a surface area of 56,600 km^2^ and a population of 8,095,498 people [14]. Overall, Togo's health system has a three-tier pyramid structure. The NMCP (that supervised this project) is located at the Central level, where policies, strategies and guidelines for malaria management are set up. The Regional (or Intermediate) level comprises six health regions and is the level that provides technical support to the Health Districts. The third (or Peripheral) level is the most decentralized; it is the operational level [15].

**Fig. 1:**
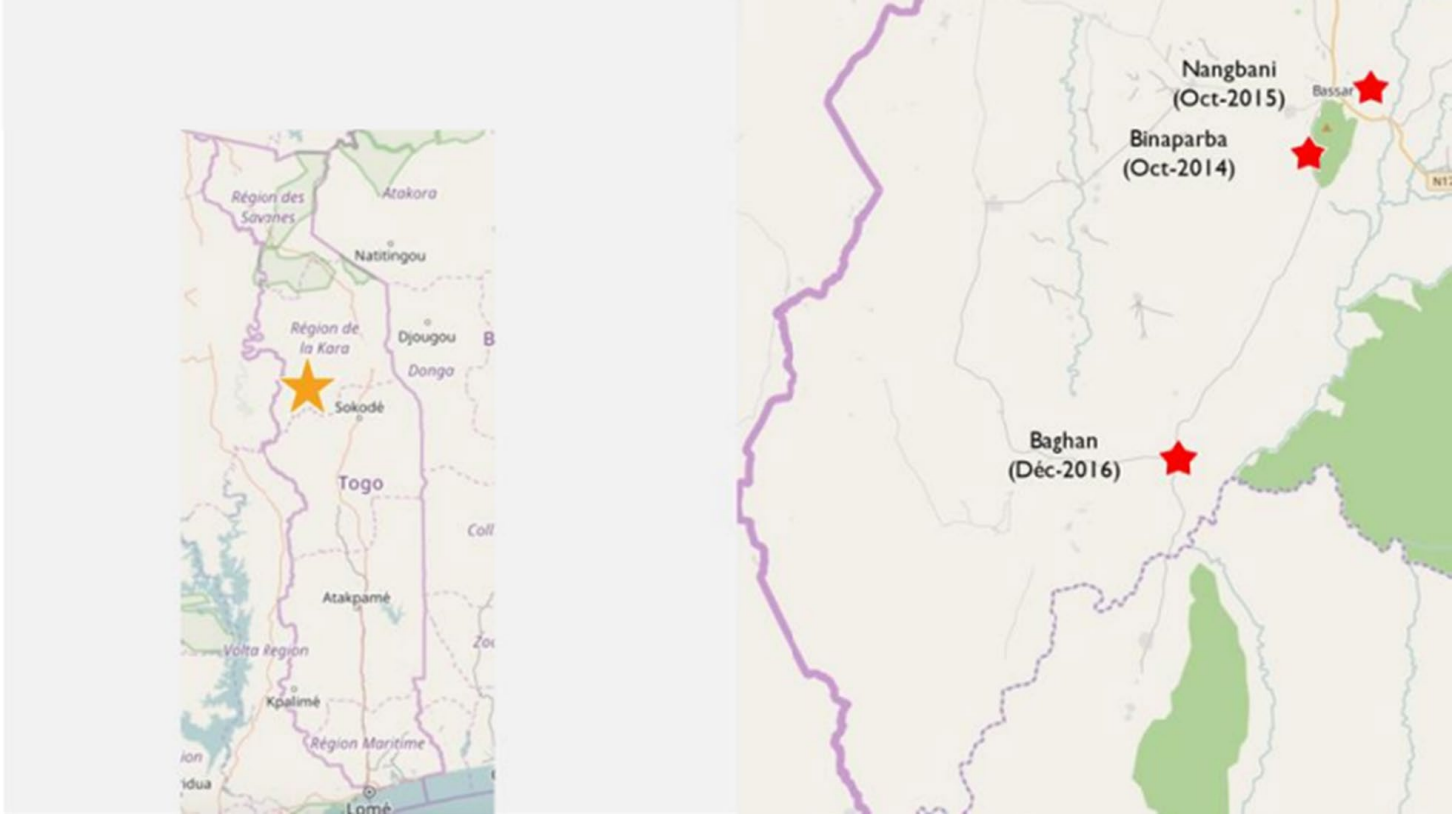
Map of the study site; i.e., Bassar District, Kara Region, Togo, 2014–2017. The red stars show the locations of the Peripheral Care Units where the PECADOM + experiment was carried out

The Bassar Health District is inhabited by 33,156 people. It comprises four communes and ten cantons that cover 3,620 km^2^. The three PCUs of Bassar where PECADOM + was implemented were: Binaparba (1303 inhabitants), Nangbani (473 inhabitants), and Baghan (4,114 inhabitants) (Fig. 1).

### Data collection

Data were collected weekly over four successive years (2014–2017) by 21 CHWs. The data collection used the following tools:

- Information-gathering media (care booklets, stock sheets);
- Report templates;
- CHW supervision grids;
- Monthly site reports;
- Supervision reports;
- Interviews using an interview guide adapted to each target group.

The information collected included data on the PCU, year, age, sex, presence of fever and malaria symptoms, RDT result, presence or absence of mosquito nets in the household, and type of detection: active (the CHWs track malaria cases in the community) or passive (the community seeks care from the CHWs or at a PCU).

In-house data checking and validation sessions were carried out to avoid missing data and identify/correct inconsistent data.

### Community health workers’ protocol

This protocol included at-home diagnosis, test, and treatment. A diagnosis of malaria required the presence of fever (hot body) or a recent history of fever during the current episode of illness without signs of severity and diagnosis confirmation by a RDT. Temperature was measured with a thermometer (axillary temperature ≥ 37.5 °C).

The RDT used in this study was SD BIOLINETM Malaria Ag P.f (HRP2/pLDH) (Standard Diagnostics Inc., Korea). This test is usually routinely used in hard-to-reach and lab-deprived areas. Its performance characteristics are: 99.3% specificity and P.f (HRP2)/P.f (pLDH) 99.7%/97.4% sensitivity.

To ensure a high testing quality, the CHWs were, first, rigorously and intensively trained in the use (preparation and interpretation) of the RDT before being sent to the villages and, second, given a quality assurance guide on how to maintain the test performance and reduce the risks of misuse.

**Fig. 2:**
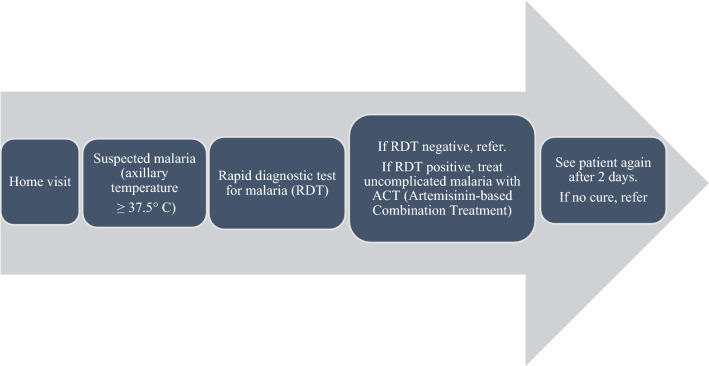
Malaria management protocol in the PECADOM + model implemented in Bassar Health District, Kara Region, Togo, 2014–2017. PCU: Peripheral care Unit. Symptoms: 0 = No symptoms, 1 = Isolated fever, 2 = Fever + headache, ache, chills, etc., 3 = Cries, dizziness

A CHW could administer ACT (using artemether-lumefantrine or artesunate-amodiaquine) to a patient with a positive RDT result. Otherwise, the patient was referred to the nearest health facility. During a follow-up visit, a CHW had to:

- Reassess the patient’s clinical condition;
- Advise on the correct use of medication;
- Emphasize preventive measures against malaria; in particular, the use of mosquito nets (Fig. 2).

### Statistical analysis

Categorical variables were described by numbers and proportions and quantitative variables by means, standard deviations, maxima, minima, and medians with interquartile ranges (IQRs). Univariate and multivariate logistic regression analyses were performed to determine the factors associated with positive RDT results. Only variables associated with a p-value ≤ 0.2 in the univariate analysis were included in the multivariate models using the bottom-up stepwise method. Interactions between the variables retained in the multivariate models were tested. The various models were then compared using the likelihood ratio test to select the more parsimonious risk model and a value of p < 0.05 was considered for statistical significance. Unadjusted and adjusted odds ratios (ORs and aORs) were reported with their 95% confidence intervals (CIs). All statistical analyses were performed using R Studio software version 14.2.2 (2022-10-31).

## Results

### Descriptive analysis

#### Data distribution by year

In all, 11,337 people participated in the study: 817 in 2014, 1,804 in 2015, 2,638 in 2016, and 6,078 in 2017. The number of participants was relatively low at the start of the project because only one PCU was included (Binaparba). This number increased after inclusion of the second and third PCUs (Nangbani then Baghan). The mean age of the participants was 18 years (SD: 16.9 years), and its median was 10 years (min–max: 0–112 years). Women accounted for 49.7% of the participants. The other participants’ characteristics are shown in Table 1.

**Table 1:** Distribution of the study variables according to the year of PECADOM + implementation in three peripheral care units in Togo

Variables	2014	2015	2016	2017	Total
	N = 817	N = 1804	N = 2638	N = 6078	N = 11,337
Peripheral care unit (PCU)					
Binaparba	817 (100%)	1362 (75.5%)	1223 (46.4%)	833 (13.7%)	4235 (37.4%)
Nangbani	–	442 (24.5%)	1061 (40.2%)	812 (13.4%)	2315 (20.4%)
Baghan	–	–	354 (13.4%)	4433 (72.9%)	4787 (42.2%)
Season					
Rainy	405 (49.6%)	897 (49.7%)	1285 (48.7%)	3992 (65.7%)	6579 (58%)
Dry	412 (50.4%)	907 (50.3%)	1353 (51.3%)	2086 (34.3%)	4758 (42%)
Age (years)					
Mean ± standard deviation	21 ± 20	19 ± 17	18 ± 16	18 ± 17	18 ± 17
Minimum–maximum	[0–80]	[0–90]	[0–97]	[0–112]	[0–112]
Median [IQR 95%]	11 [5–35]	12 [5–32]	10 [5–29]	9 [4–28]	10 [5–29]
Age category (years)					
[0–1]	42 (5.1%)	46 (2.5%)	72 (2.7%)	287 (4.7%)	447 (3.9%)
[1–2]	35 (4.3%)	86 (4.8%)	129 (4.9%)	473 (7.8%)	723 (6.4%)
[2–3]	62 (7.6%)	104 (5.8%)	153 (5.8%)	452 (7.4%)	771 (6.8%)
[3–4]	53 (6.5%)	136 (7.5%)	155 (5.9%)	387 (6.4%)	731 (6.4%)
[4–5]	57 (7%)	139 (7.7%)	163 (6.2%)	448 (7.4%)	807 (7.1%)
[5–10]	143 (17.5%)	326 (18.1%)	657 (24.9%)	1194 (19.6%)	2320 (20.5%)
[10–15]	67 (8.2%)	144 (8%)	255 (9.7%)	494 (8.1%)	960 (8.5%)
[15–25]	66 (8.1%)	209 (11.6%)	297 (11.3%)	676 (11.1%)	1248 (11%)
> 25	292 (35.7%)	614 (34%)	757 (28.7%)	1667 (27.4%)	3330 (29.4%)
Sex					
Female	418 (51.2%)	896 (49.7%)	1347 (51.1%)	2976 (49%)	5637 (49.7%)
Male	399 (48.8%)	908 (50.3%)	1291 (48.9%)	3102 (51%)	5700 (50.3%)
Symptoms					
No symptoms	596 (72.9%)	868 (48.1%)	1383 (52.4%)	4046 (66.6%)	6893 (60.8%)
Isolated fever	98 (12%)	467 (25.9%)	559 (21.2%)	743 (12.2%)	1867 (16.5%)
Fever + headache, ache, chills, etc	32 (3.9%)	26 (1.4%)	157 (6%)	799 (13.1%)	1014 (8.9%)
Cries/dizziness	91 (11.1%)	443 (24.6%)	539 (20.4%)	490 (8.1%)	1563 (13.8%)
Rapid diagnostic test result					
Positive	580 (72.2%)	1364 (76.2%)	1945 (74%)	3689 (62.1%)	7578 (67.9%)
Negative	223 (27.8%)	427 (23.8%)	683 (26%)	2254 (37.9%)	3587 (32.1%)
Not done	14	13	10	135	172
Presence of mosquito net					
Yes	–	–	623 (87.3%)	4370 (77.5%)	4993 (78.6%)
No	–	–	91 (12.7%)	1266 (22.5%)	1357 (21.4%)
Unknown	817	1804	1924	442	4987
Detection type					
Active	385 (61.3%)	763 (47.5%)	1226 (49.5%)	4368 (73.7%)	6742 (63.4%)
Passive	243 (38.7%)	842 (52.5%)	1251 (50.5%)	1555 (26.3%)	3897 (36.6%)
Undetermined	189	199	161	155	704

#### Data distribution by PCU

The mean age of the participants was 20 years in Binaparba (median: 12; min–max: 0–112), 17 years in Nangbani (median: 10; min–max: 0–90) and 16 years in Baghan (median: 9; min–max: 0–112). According to the RDT results, people who tested positive represented 75.3% of the participants in Binaparba, 77.4% in Nangbani, and 56.6% in Baghan. Household mosquito net ownership was 82% in Binaparba, 99.1% in Nangbani, and 73.7% in Baghan. Regarding the type of detection, the active approach allowed CHWs to detect 38.1% of malaria cases in Binaparba, 68.8% in Nangbani, and 80.6% in Baghan. Other interesting data are given in Table 2.

**Table 2:** Distribution of the study variables according to each Peripheral Care Unit (Kara Region, Togo, 2014 to 2017)

Variables	Binaparba	Nangbani	Baghan
	N = 4235	N = 2315	N = 4787
Season			
Rainy	2293 (54.1%)	1,336 (57.7%)	2950 (61.6%)
Dry	1942 (45.9%)	979 (42.3%)	1837 (38.4%)
Age (years)			
Mean ± Standard deviation	20 ± 18	17 ± 15	16 ± 17
Minimum–maximum	[0–112]	[0–90]	[0–112]
Median [IQR 95%]	12 [6–32]	10 [5–26]	9 [4–27]
Age category (years)			
[0–1]	105 (2.5%)	76 (3.3%)	266 (5.6%)
[1–2]	182 (4.3%)	134 (5.8%)	407 (8.5%)
[2–3]	227 (5.4%)	158 (6.8%)	386 (8.1%)
[3–4]	247 (5.8%)	127 (5.5%)	357 (7.5%)
[4–5]	290 (6.8%)	140 (6%)	377 (7.9%)
[5–10]	860 (20.3%)	591 (25.5%)	869 (18.2%)
[10–15]	397 (9.4%)	233 (10.1%)	330 (6.9%)
[15–25]	470 (11.1%)	274 (11.8%)	504 (10.5%)
> 25	1291 (27%)	1457 (34.4%)	582 (25.1%)
Sex			
Female	2122 (50.1%)	1192 (51.5%)	2323 (48.5%)
Male	2113 (49.9%)	1123 (48.5%)	2464 (51.5%)
Symptoms			
No symptoms	2227 (52.6%)	1330 (57.5%)	3336 (69.7%)
Isolated fever	887 (20.9%)	477 (20.6%)	503 (10.5%)
Fever + headache, ache, chills, etc	215 (5.1%)	153 (6.6%)	646 (13.5%)
Cries/dizziness	906 (21.4%)	355 (15.3%)	302 (6.3%)
Rapid diagnostic test result			
Positive	3151 (75.3%)	1786 (77.4%)	2641 (56.6%)
Negative	1036 (24.7%)	522 (22.6%)	2029 (43.4%)
Not done	48	7	117
Presence of mosquito net			
Yes	860 (82%)	887 (99.1%)	3246 (73.7%)
No	189 (18%)	8 (0.9%)	1160 (26.3%)
Unknown	3186	1420	381
Detection type			
Active	1397 (38.1%)	1557 (68.8%)	3788 (80.6%)
Passive	2274 (61.9%)	705 (31.2%)	912 (19.4%)
Undetermined	564	53	87

#### Distribution of RDT results

Overall, the proportion of positive RDTs was 62% during the rainy season and 38% during the dry season. A decrease in positive RDTs was observed by the end of each rainy season.

The mean age at malaria case detection by RDT was 16 years (median: 9; min–max: 0–11). However, the 5–10 age group was the most affected; it represented 24.2% of all positive RDTs. Although 85% of the households in this study had mosquito nets, 77.2% of the RDTs were positive. Data relative to other variables linked with RDT results are given in Table 3.

**Table 3:** Distribution of the study variables according to the results of the rapid diagnostic test for malaria in Bassar Health District, Kara Region, Togo, 2017–2017

Variables	Positive RDT	Negative RDT
	N = 7578	N = 3587
Season		
Rainy	4700 (62%)	1839 (51.3%)
Dry	2878 (38%)	1748 (48.7%)
Age (years)		
Mean ± standard deviation	16 ± 16	22 ± 18
Minimum–maximum	[0–11]	[0–112]
Median [IQR 95%]	9 [4–25]	18 [5–35]
Age category (years)		
[0–1]	283 (3.7%)	161 (4.5%)
[1–2]	492 (6.5%)	228 (6.4%)
[2–3]	556 (7.3%)	212 (5.9%)
[3–4]	539 (7.1%)	186 (5.2%)
[4–5]	601 (7.9%)	200 (5.6%)
[5–10]	1834 (24.2%)	471 (13.1%)
[10–15]	697 (9.2%)	248 (6.9%)
[15–25]	766 (10.1%)	454 (12.7%)
> 25	1810 (23.9%)	1427 (39.8%)
Sex		
Female	3691 (48.7%)	1870 (52.1%)
Male	3887 (51.3%)	1717 (47.9%)
Symptoms		
No symptoms	4145 (54.7%)	2626 (73.2%)
Isolated fever	1570 (20.7%)	292 (8.1%)
Fever + headache, ache, chills, etc	673 (8.9%)	338 (9.4%)
Cries/dizziness	1190 (15.7%)	331 (9.2%)
Presence of mosquito net		
Yes	3043 (77.2%)	1992 (81.3%)
No	897 (22.8%)	442 (18.7%)
Unknown	3638	1223
Detection type		
Active	4149 (58.6%)	2519 (72.8%)
Passive	2928 (41.4%)	942 (27.2%)
Undetermined	501	126

### Results of the univariate logistic regression analysis

The univariate logistic regression model showed that the probability of testing positive was 1.55 times higher during the rainy than during the dry season (OR = 1.55; 95% CI 1.43–1.68).

Men appeared to be 1.15 times more likely to have positive RDTs than women (OR = 1.15; 95% CI 1.06–1.24) and people with fever (≥ 37.5 °C) were significantly more likely to have positive RDTs than asymptomatic people (OR = 3.41; 95% CI 2.98–3.90). When fever was associated with other malaria symptoms, the probability of positive RDT was 1.26 times higher (OR = 1.26; 95% CI 1.10–1.45) than in the absence of association. Without fever but in the presence of other symptoms, the probability of testing positive was multiplied by 2.28 (OR = 2.28; 95% CI 2.00–2.60).

In passive case detection, the risk of a positive RDT was 1.89 times higher than in active detection (OR = 1.89; 95% CI 1.73–2.0). The results of the univariate analysis are shown in Table 4.

**Table 4:** Results of the univariate analysis of factors associated with positive rapid diagnostic test result for malaria in Bassar Health District, Kara Region, Togo, 2014–2017

Variables	Raw OR	[CI 95%]	P-value
Year			
2014	Reference		
2015	1.23	[1.02–1.48]	0.033
2016	1.1	[0.92–1.31]	0.316
2017	0.63	[0.53–0.74]	< 0.001
Season			
Dry	Reference		
Rain	1.55	[1.43–1.68]	< 0.001
Peripheral care unit			
Binaparba	Reference		
Baghan	0.43	[0.39–0.47]	< 0.001
Nangbani	1.13	[0.99–1.27]	0.055
Age category (Year)			
[0–1]	Reference		
[1–2]	1.23	[0.96–1.58]	0.120
[2–3]	1.49	[1.16–1.92]	0.002
[3–4]	1.65	[1.28–2.13]	< 0.01
[4–5]	1.71	[1.33–2.20]	< 0.01
[5–10]	2.22	[1.78–2.75]	< 0.01
[10–15]	1.60	[1.26–2.04]	< 0.01
[15–25]	0.96	[0.77–1.20]	0.722
> 25	0.72	[0.58–0.89]	0.002
Sex			
Female	Reference		
Male	1.15	[1.06–1.24]	< 0.001
Symptoms			
No symptoms	Reference		
Isolated fever	3.41	[2.98–3.90]	< 0.001
Fever + headache, ache, chills, etc.	1.26	[1.10–1.45]	0.001
Cries/dizziness	2.28	[2.00–2.60]	< 0.001
Detection			
Active	Reference		
Passive	1.89	[1.73–2.06]	< 0.001

### Results of the multivariate regression analysis

These results showed that, vs. Binaparba, the probability of positive RDT was significantly lower in Baghan (aOR = 0.53; 95% CI 0.47–0.59) and higher in Nangbani (aOR = 1.30; 95% CI 1.14–1.49). During the rainy season, the probability of testing positive appeared to be 1.76 times higher than during the dry season (aOR = 1.74; 95% CI 1.60–1.90).

Regarding age, there was no significant difference in the probability of positivity between children aged 0–1 years and those aged 1–2 years. However, from age 2 up to 15, the likelihood of a positive test was, on average, 1.52 times higher than in the reference age group (0 to one year). This probability decreased significantly after age 25 (aOR = 0.65; 95% CI 0.52–0.81). In terms of sex, the multivariate analysis revealed an association between being a male and the probability of testing positive (aOR = 1.11; 95% CI 1.02–1.21).

Having a fever (≥ 37.5 °C) significantly increased the probability of a positive test (aOR = 2.19; 95% CI 1.89–2.54). This increase was also observed for symptoms other than fever (aOR = 1.83; 95% CI 1.59–2.12). Also, the presence of fever along with other symptoms appeared to be associated with positive malaria detection (aOR = 1.16; 95% CI 1.00–1.35); however, this association was not significant (the CI of the aOR included value 1).

**Fig. 3:**
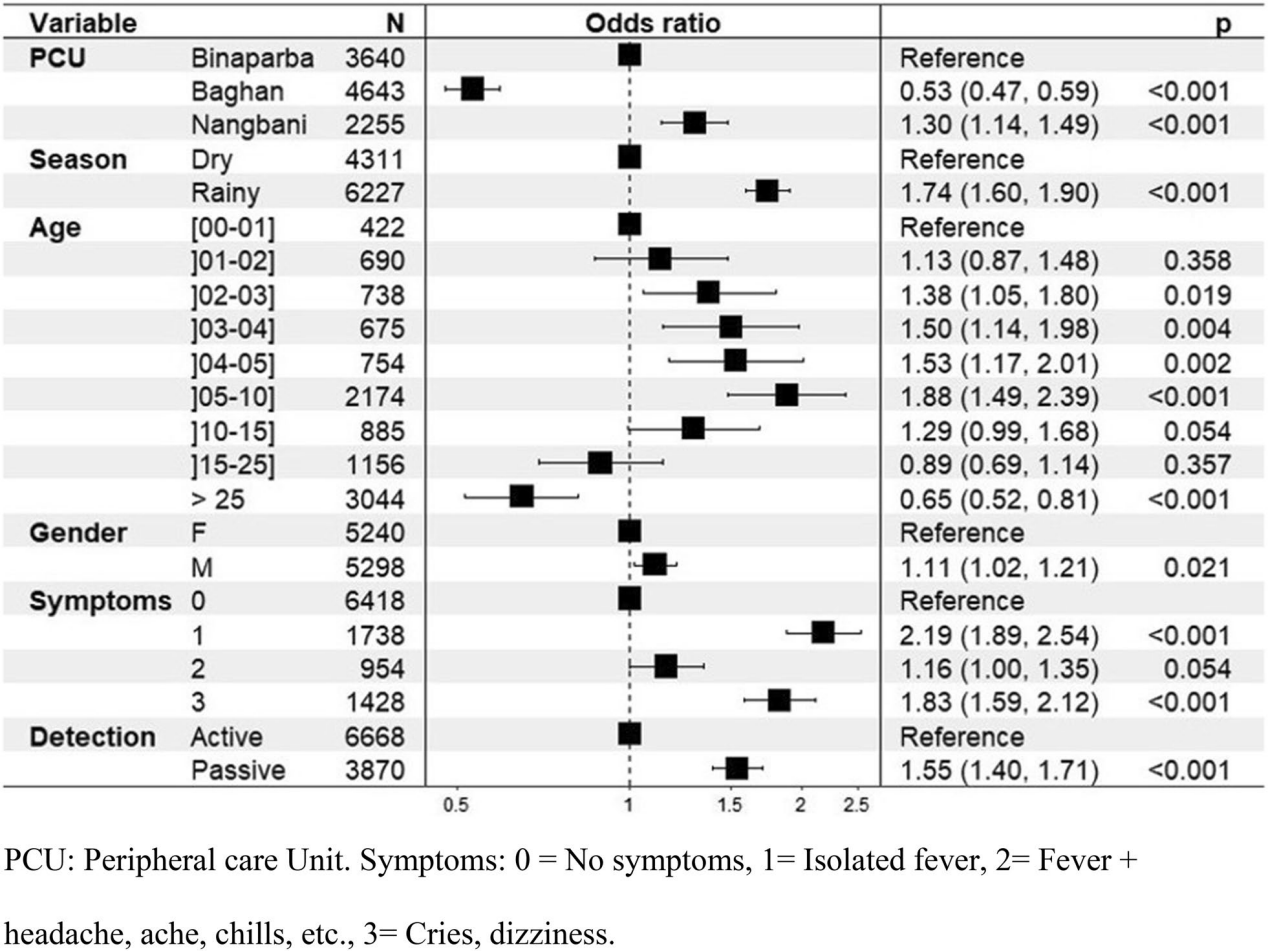
Forest plot resulting from the multivariate regression analysis of the factors associated with positive rapid test results for malaria in Bassar Health District, Kara Region, Togo, 2014–2017

Passive case detection was found to be associated with a higher probability of positive RDT result than active case detection (aOR = 1.55; 95% CI 1.40–1.71). The results of the multivariate analysis are shown in Fig. 3.

## Discussion

In Togo, as in other malaria-affected countries, early detection of cases is of crucial importance for adequate care and optimal management of the disease as part of its eradication [16–18]. PECADOM + , as other CHW-led projects, aimed to improve access to malaria screening, treatment, and management services to reduce disease complications (especially in children and pregnant women), reduce transmission in remote communities, and accelerate eradication [19]. This intervention aligns with the WHO's vision of eradicating malaria worldwide by 2030 [19]. As per the study design and the WHO recommendations for such an initiative, the objective of the CHWs was not to test all household members but to identify only individuals suspected of having malaria (current or recent fever and positive RDT) so they could be rapidly evaluated and treated.

Experimenting PECADOM + in Togo highlighted differences in the risk of positive RDT between the participating PCUs. This suggests that the way of implementing community management of malaria may differ according to local characteristics such as demography, behavior, and type of intervention.

Also, the results highlighted differences in transmission and risk between seasons (rainy vs. dry), indicating the importance of a more sustained screening during the rainy season in response to the favorable conditions for mosquito vector reproduction [20]. This result is in line with recent studies conducted in Togo. Indeed, a survey of the trends in malaria morbidity and mortality in Togo from 2008 to 2017 showed that morbidity tended to increase throughout the country during the rainy seasons despite intensification of various strategies and interventions [15]. Similarly, a study conducted on chemoprevention of seasonal malaria in Togo from 2013 to 2020 reported that, before implementing this intervention, frequent increases of malaria cases were seen during the rainy seasons, especially among children under five [21]. Finally, a study conducted from 2008 to 2017 in Togo showed that maximum seasonal indices were seen during rainy seasons and minimum seasonal indices during dry seasons [22].

The results of the present experiment showed that children aged five to ten years were the most at risk of malaria. According to internal NMCP reports and a significant number of other sources, children under five are generally the most vulnerable to malaria. Here, the trend was a shift in morbidity towards slightly older children. This might be linked to the effect of implementing the Seasonal Malaria Chemoprevention (SMC) strategy in children aged 3–59 months during seasons of high malaria transmission in Bassar Health District. In some countries of the West African sub-region (Senegal, Mali), this observation has led to reconsidering SMC targets and extending them to children under ten [23–25].

One expected result was that fever was often present in RDT-positive subjects. This confirms that fever is a crucial symptom of malaria. However, other symptoms, such as headache, body aches, chills, dizziness, and tiredness, were also associated with positive RDT results. In an early detection approach, it is necessary to pay attention to the latter symptoms. This would help early detections and rapid treatments that save lives.

Mosquito nets are known to be efficient in reducing the risk of being bitten by malaria mosquito vectors. Therefore, mosquito nets in households are necessary to prevent malaria transmission. Within this experiment, nets were present in nearly all visited households; however, it was impossible to evaluate their exact impact because no information was collected regarding their proper use.

Finally, this experiment indicated that passive detection would increase the probability of finding positive tests vs. active home visits by CHWs. This contrasts with results obtained by Linn et al. [12] showing that the prevalence of RDT-confirmed malaria was 16 times higher in passively-screened than in actively-screened villages. One explanation is that passively-detected malaria cases in this experiment were genuine cases; sick people would come more readily to the CHWs in the villages when they feel ill. The increase in the probability of finding positive tests through active home visits underlines the importance of active case detection in the communities through regular screening and targeting specific or at-risk groups. Active home visits also help case identification before developing clinical signs; thus, early management. However, more information is needed on how the CHWs carried out the two types of detection; this will optimize the detection strategy and improve the quality of care.

The literature still needs to report more on the role of CHWs in the fight against malaria [26–30]. In Africa, most studies were carried out in West Africa and East sub-Saharan Africa [31–33]. The present study is most probably the first in Togo to describe community management of malaria and identify factors associated with positive RDT results. It presents a major asset of an exhaustive participation of the PCUs of Bassar Health District between 2014 and 2017.

It is worth mentioning that, among the strategies already used by the NMCP to combat malaria in Togo, PECADOM + is not yet adopted. At the time of this study, it was only experimented in a few PCUs in Bassar Health District (Kara Region) before a more extended implementation. Thus, this experimentation’s results cannot be generalized to the entire Bassar Health District, entire Kara Region, or entire Togo.

The results obtained here are interesting and place Togo among the countries that have experimented with this innovative strategy [12, 35, 36]. These results are similar to those found in the scientific literature. Indeed, in Togo, the CHWs detected more malaria cases in intervention villages [36]. Based on these results, the NMCPs of the countries that experimented with this new approach were able to provide workable guidelines to improve the implementation of community management of malaria, as well as guidance for other NMCPs that consider adopting it.

To be properly implemented and maintained, Togo's PECADOM + needs to be adequately funded to be correctly implemented and maintained. Given the financial difficulties already met in carrying out this experiment, one may expect major problems in obtaining funds for the programme extension to whole regions.

## Conclusion

PECADOM + is a proactive community-based malaria prevention and control strategy that is strongly recommends by the WHO. In Togo, it is currently in an experimental phase before implementation across the whole country. The present experiment showed that important measures should be taken to improve its implementation and follow-up. These include adopting an active targeted approach to malaria case detection, optimizing data collection, checking the reproducibility of the current implementation conditions in other Health Districts, and, finally, overcoming all financial challenges by mobilizing domestic resources and lobbying all possible funders.

## Data Availability

The datasets generated and/or analysed during the current study are not publicly available due to their belonging to the Togolese Ministry of Health and Public Hygiene but are available from the corresponding author on reasonable request.

## Abbreviations

ACT: Artemisinin-based combination therapy
AOR: Adjusted odds ratio
CHW: Community health worker
CI: Confidence interval
COVID: Coronavirus infection disease
IQR: Interquartile range
NMCP: National Malaria Control Programme
OR: Odds ratio
PCU: Peripheral care unit
RDT: Rapid diagnostic test
SD: Standard deviation
SMC: Seasonal malaria chemoprevention
UNICEF: United Nations Children's Fund
WHO: World Health Organization
